# *Calmodulin-Like (CML)* Gene Family in *Medicago truncatula*: Genome-Wide Identification, Characterization and Expression Analysis

**DOI:** 10.3390/ijms21197142

**Published:** 2020-09-27

**Authors:** Qiguo Sun, Shuhan Yu, Zhenfei Guo

**Affiliations:** College of Grassland Science, Nanjing Agricultural University, Nanjing 210095, China; 2017220007@njau.edu.cn (Q.S.); 2018220001@njau.edu.cn (S.Y.)

**Keywords:** CML, *Medicago truncatula*, expression profiling, promoter *cis*-acting element, abiotic stress

## Abstract

Calcium is an important second messenger in mediating adaptation responses of plants to abiotic and biotic stresses. Calmodulin-like (CML) protein is an important calcium-signaling protein that can sense and decode Ca^2+^ signal in plants. *Medicago truncatula* is a model legume plant; however, investigations of MtCML proteins are limited. Using genome analysis and BLAST database searches, fifty MtCML proteins that possess EF-hand motifs were identified. Phylogenetic analysis showed that CML homologs between *M. truncatula*, *Arabidopsis thaliana* and *Oryza sativa* shared close relationships. Gene structure analysis revealed that these *MtCML* genes contained one to four conserved EF-hand motifs. All *MtCMLs* are localized to eight chromosomes and underwent gene duplication. In addition, *MtCML* genes were differentially expressed in different tissues of *M. truncatula*. *Cis*-acting elements in promoter region and expression analysis revealed the potential response of MtCML protein to abiotic stress and hormones. The results provide a basis of further functional research on the *MtCML* gene family and facilitate their potential use for applications in the genetic improvement on *M. truncatula* in drought, cold and salt stress environments.

## 1. Introduction

Calcium is a universal second messenger in mediating adaptation responses of plants to abiotic and biotic stresses [1]. Diverse stimuli such as plant hormones, low temperature, drought, salt, and pathogens induce rapid and transient changes in cellular Ca^2+^ concentration [2], which are sensed and decoded by calcium-binding proteins (CBP) to activate downstream reactions [3]. The CBPs include calmodulins (CaMs), CaM-like proteins (CMLs), Ca^2+^-dependent protein kinases (CPKs/CDPKs) and calcineurin B-like proteins (CBLs) in plants [4,5,6]. A typical CaM contains four EF-hand motifs [7], while an EF-hand motif is composed of two helices, the E helix and the F helix, flanking a Ca^2+-^binding loop in a structure that resembles a hand [8]. CMLs are a unique class of EF-hand proteins and specifically existed in plants. The *CML* gene family has been characterized in some plant species, such as *Arabidopsis* [9], rice [10], grapevine (*Vitis amurensis*) [11], Chinese cabbage *(Brassica rapa)* [12] and tomato (*Solanum lycopersicum*) [13].

CMLs regulate downstream targets in response to various stimuli-induced Ca^2+^ fluctuations and signal transduction [14]. In *Arabidopsis*, CML15 and CML16 show changes in electrophoretic mobility in the presence of Ca^2+^, which confirms their ability to bind Ca^2+^ [15,16]. The roles of CMLs in plant development and stress responses were investigated [17,18,19]. CML24 regulates root mechanoresponses, pollen tube growth and flowering in *Arabidopsis* [20,21,22,23]. CML24 can respond circadian oscillations of cytosolic-free calcium and regulate the circadian clock [24]. The other CMLs, such as CML12, CML25, CML38, CML39 and CML43, are involved in plant growth and development [25,26,27,28,29]. CML8, CML41, CML42, CML46 and CML47 are involved in plant resistance to bacterium pathogens [30,31,32,33], while *CML9*, *CML24*, *CML37*, *CML38*, and *CML39* transcripts are induced by salt or/and drought stress [2,23,34,35]. CML20 is a negative regulator, while CML24 and CML37 are positive regulators in ABA-signaling during drought stress [23,34,36]. OsCML16, a direct target of OsERF48, appears to transduce OsERF48 actions to downstream target genes that together confer the acquired root phenotype and drought tolerance in rice [37]. GsCML27 is a positive regulator of plant tolerance to bicarbonate stress, but a negative regulator of salt or osmotic stresses during early growth stages in *Glycine soja* [38]. CML proteins play crucial roles in diverse physiological processes.

*M. truncatula* is an important model leguminous plant [39]. MtCML40 is a negative regulator of salt tolerance in *M. truncatula* by downregulating *MtHKTs* expression and leading to Na^+^ toxicity [40]. However, the other MtCMLs have not been investigated. The objective of this study was to perform a genome-wide analysis of CMLs in *M. truncatula* including gene structure, chromosomal location, duplication, EF-hand motif organization and expression characteristics. The results provide a comprehensive understanding of the *CML* gene family in *M. truncatula*.

## 2. Results

### 2.1. Identification of CML Members in M. truncatula

A genome-wide search for *CML* genes was performed using the BLASTP program based on the completed genome sequence of *M. truncatula*, using *Arabidopsis* and rice *CML* genes as the query sequences. Fifty *MtCML* genes were obtained and their deduced peptides were subjected to domain analysis using Pfam and SMART databases for further confirmation. A total of 50 members were identified, and they were named as *MtCML1* to *MtCML50* (Table 1). The amino acid sequence of MtCMLs was further analyzed. MtCMLs had an average of approximately 167 amino acids in length, ranging from 65 amino acids in MtCML28 to 266 amino acids in MtCML11. The predicted molecular weight varied from 7.37 kDa to 29.98 kDa, and the theoretical isoelectric point (pI) varied from 4.01 to 9.01 (Table 1). The predicted grand average of hydropathicity (GRAVY) of all MtCMLs was negative, indicating that MtCMLs are hydrophilic proteins (Table 1).

### 2.2. Phylogenetic Relationship of CMLs among M. truncatula, Arabidopsis and Rice

**Figure 1:**
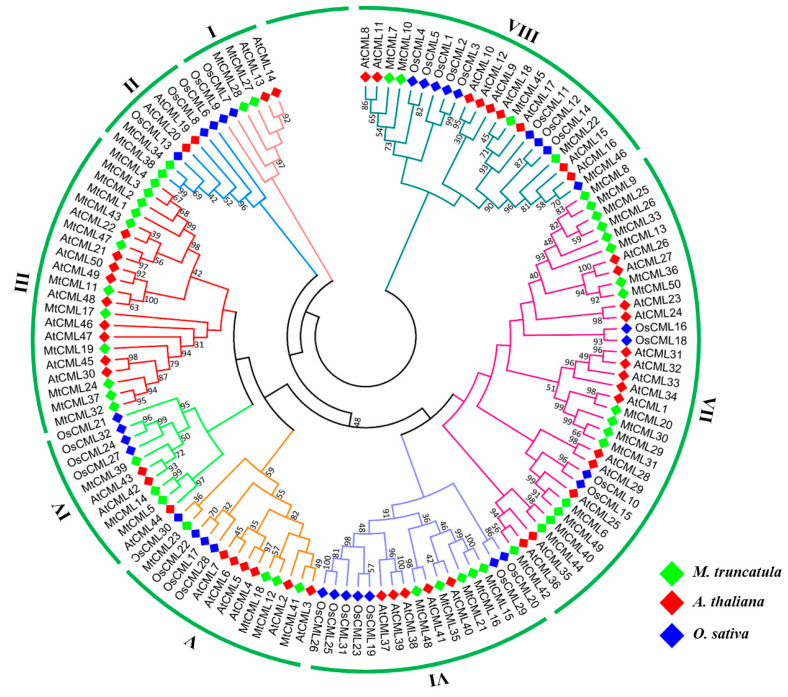
Phylogenetic tree of calmodulin-like (CML) proteins in *Medicago truncatula*, *Arabidopsis thaliana* and *Oryza sativa.* Tree constructed with 1000 bootstrap replications. CMLs from *M. truncatula*, *A. thaliana* and *O. sativa* distinguished using green, red and blue color, respectively.

To evaluate the evolutionary relationship, all CML members from *M. truncatula* (50), *Arabidopsis* (50) and rice (32) were aligned using the maximum-likelihood (ML) method to generate an unrooted phylogenetic tree (Figure 1). The sequences of MtCML proteins are shown in Table S1. CML proteins in these species could be clustered into eight groups (Groups 1–8). The results suggest existence of a common ancestor before the divergence of monocots and dicots. More members were grouped in Group 3, 6, 7 and 8 than in others. Group 3 contained *Arabidopsis* and *M. truncatula* CMLs members, but not rice CMLs (Figure 1), indicating that there are differences in the evolution of CML proteins between monocots and dicots. In addition, most of the MtCML showed homologous to those in *Arabidopsis* and rice (Figure 1). The results indicated that CMLs are conserved among plant species.

### 2.3. Gene Structure and Domain Architectures

**Figure 2:**
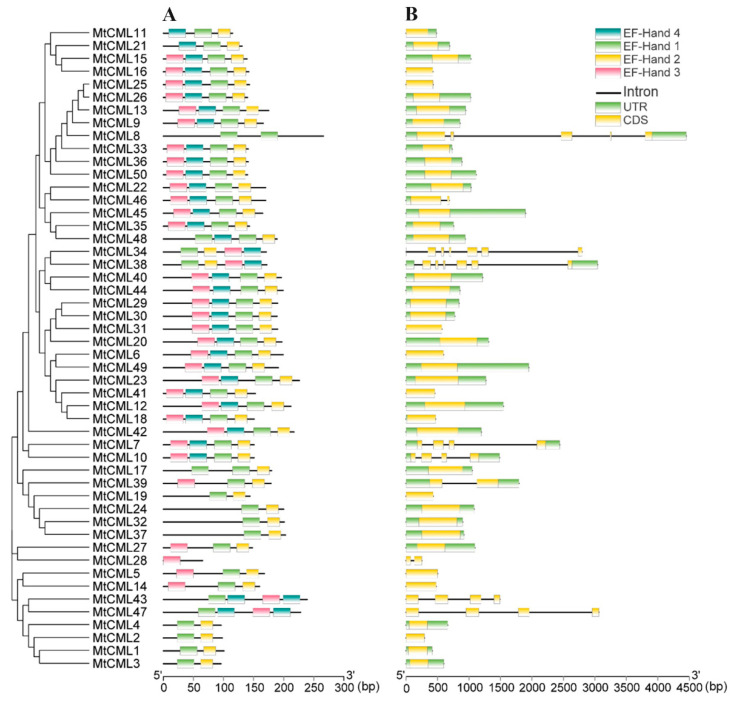
Characterization of *MtCML* genes and MtCML proteins. (**A**) EF-hand motifs; (**B**) exon–intron structure distribution.

*MtCMLs* structure was analyzed based on the arrangement of exon/intron. No intron was found in 40 members of *MtCMLs*, while one to six introns were found in the others. One intron was found in three *MtCMLs* (*MtCML28*, *MtCML39* and *MtCML46*); three introns in four *MtCMLs* (*MtCML7*, *MtCML10*, *MtCML43* and *MtCML47*), four in *MtCML11* and six in *MtCML34* and *MtCML38* (Figure 2A).

EF-hand motifs are the most prominent structural feature in CML proteins. Most of MtCML members (33) contained four conserved EF-hand motifs, and seven members contained three EF-hand motifs. Two EF-hand motifs were present in nine MtCML members (MtCML1, MtCML2, MtCML3, MtCML4, MtCML11, MtCML19, MtCML24, MtCML32 and MtCML27), while one EF-hand motif in MtCML28 (Figure 2B).

### 2.4. Chromosomal Location and Synteny Analysis of MtCML Genes

**Figure 3:**
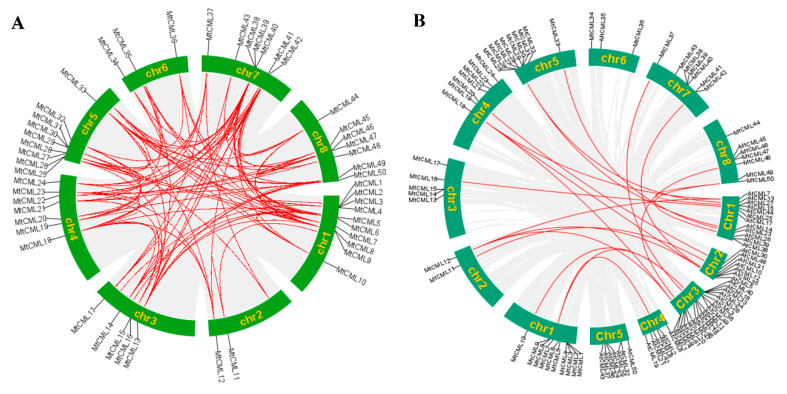
Chromosomal distribution and synteny analysis of *CML* genes in the genomes of *Medicago truncatula* and *Arabidopsis thaliana*. (**A**) Paralogous *MtCML* genes mapped onto *M. truncatula* chromosomes; (**B**) Orthologous *CML* genes mapped onto *M. truncatula* (chromosomes 1–8) and *A. thaliana* (chromosomes 1–5). Red lines indicate duplicated *MtCML* gene pairs.

*MtCMLs* were mapped onto the chromosomes against the *M. truncatula* genome database to examine their chromosomal distribution. *MtCML*s were located on chromosome 1 to 8. Ten *MtCMLs* (*MtCML1*, *MtCML2*, *MtCML3*, *MtCML4*, *MtCML5*, *MtCML6*, *MtCML7*, *MtCML8*, *MtCML9* and *MtCML10*) were located on chromosome 1, while nine (*MtCML25*, *MtCML26*, *MtCML27*, *MtCML28*, *MtCML29*, *MtCML30*, *MtCML31*, *MtCML32* and *MtCML33*) on chromosome five. There were seven *MtCMLs* locating on chromosomes four, seven and eight, respectively. In addition, five *MtCML*s (*MtCML13*, *MtCML14*, *MtCML15*, *MtCML16* and *MtCML17*) were located on chromosome three, three (*MtCML34*, *MtCML35* and *MtCML36*) on chromosomes six and two (*MtCML11* and *MtCML12*) on chromosome two (Figure 3A). The results indicated that *MtCML*s were randomly scattered on different chromosomes.

Collinearity diagrams among *MtCML*s were further analyzed. The results showed that all *MtCML* genes underwent gene duplication in *M. truncatula* genome (Figure 3A, Table S7). Most *MtCML* genes were found with 1–3 paralogous genes in *M. truncatula.* Five *MtCML* genes (*MtCML5*, *MtCML6*, *MtCML8*, *MtCML12* and *MtCML20*) had four homologous genes in *M. truncatula.* In addition, a synteny analysis of *CML* genes among *M. truncatula* and *A. thaliana* was performed. Twenty pairs of orthologous CMLs were identified between *M. truncatula* and *A. thaliana* (Figure 3B, Table S7). Three genes (*MtCML7*, *MtCML27* and *MtCML50)* had two homologous genes in *A. thaliana*, while two genes (*MtCML12* and *MtCML18)* had three orthologous genes in *A. thaliana*. The percentage of identity between pairs of paralogous MtCML proteins ranged from 21.69% to 100% in *M. truncatula* (Table S7). Among *M. truncatula* and *A. thaliana*, the identity between pairs of orthologous ranged 28.06% to 87.16%. Some high percentages of identity between MtCMLs and AtCMLs suggest that MtCML protein sequences and functions could be highly conserved. These results indicated that *MtCML* genes may be generated by gene duplication, which may be associated with conserved amino acid sequence in MtCML.

### 2.5. Spatial and Temporal Expression Profiles of MtCMLs

**Figure 4:**
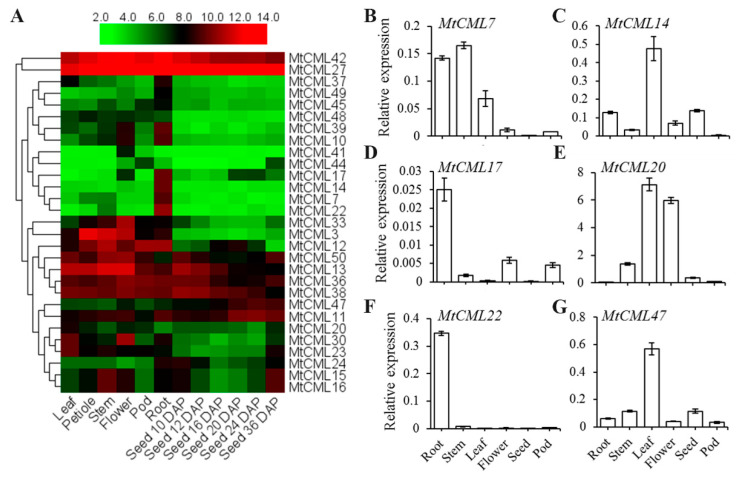
Expression analysis of *MtCML* genes in different organs. (**A**) Expression patterns of *MtCML* genes in leaves, petioles, stems, flowers, pods, roots and seeds. The expression levels of *MtCML* genes are shown as the log2-based fluorescence intensity values from the microarray data (MtGEA, https://mtgea.noble.org/v3/). DAP, days after pollination; (**B**–**G**) relative expression of (**B**) *MtCML7*; (**C**) *MtCML14;* (**D**) *MtCML17;* (**E**) *MtCML20;* (**F**) *MtCML22;* (**G**) *MtCML47* in roots, stems, leaves, flowers, seeds and pods of *Medicago truncatula.* Expression data obtained via qRT-PCR. *MtActin* used as an internal control in the qRT-PCR experiments.

The spatial and temporal expression profiles of *MtCMLs* were examined based on the microarray data from *M. truncatula* Gene Expression Atlas (MtGEA, https://mtgea.noble.org/v3/). Twenty-nine *MtCML*s have corresponding probe sets in the dataset (Table S2). *MtCML13*, *MtCML27*, *MtCML36*, *MtCML38* and *MtCML42* exhibited relatively high expression in all tissues (flowers, leaves, petioles, pods, stems, roots and seeds) (Figure 4A), indicating that their functions may be extensive. *MtCML7*, *MtCML14*, *MtCML17* and *MtCML22* were preferentially expressed in roots, and *MtCML47* was only expressed in mature seeds, while *MtCML20* and *MtCML30* were expressed in leaves. Some *MtCMLs* (*MtCML6*, *MtCML13*, *MtCML25*, *MtCML28* and *MtCML30*) were highly expressed in floral organs (Figure 4A). On the other hand, relative expression of *MtCML7*, *MtCML14*, *MtCML17*, *MtCML20*, *MtCML22* and *MtCML47* was examined using qRT-PCR (Figure 4B–G)*. MtCML7* was mostly expressed in roots, stems and leaves, but not in seeds (Figure 4B); *MtCML14* was highly expressed in leaves and its transcript could be detected in roots, stems, flowers and seeds (Figure 4C). *MtCML17* was highly expressed in roots, and its transcript could be detected in stems, flowers and pods (Figure 4D). *MtCML20* was major expressed in leaves and flowers as well as in stems, but not in roots and pods (Figure 4E), *MtCML22* was only highly expressed in roots (Figure 4E). *MtCML47* was expressed in all detected organs with highest transcript in leaves (Figure 4F). The expression patterns of *MtCML7*, *MtCML17*, *MtCML20*, *MtCML22* and *MtCML47* were consistent with that in microarray data. The different spatial and temporal expression patterns of the *MtCML* genes suggest their functional diversity in *Medicago* development.

### 2.6. Analysis of cis-Acting Element in the Promoter Region of MtCML Genes

**Figure 5:**
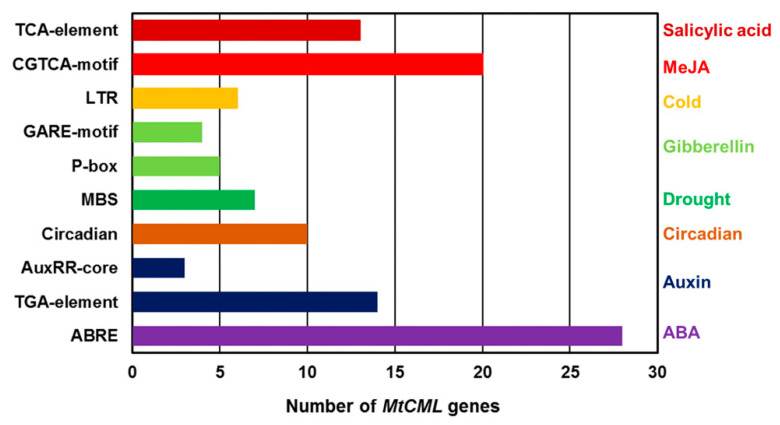
Number of *MtCML* genes containing various *cis*-acting elements. ABRE, ABA-responsive element. LTR—*cis*-acting element involved in low-temperature response; CGTCA-motif—*cis*-acting element involved in MeJA response; TCA-element—*cis*-acting element involved in SA response; GARE-motif and *P-*box—*cis*-acting element involved in GA response; MBS—*cis*-acting element involved in drought response; circadin—*cis*-acting element involved in biologic rhythms; AuxRR-core and TGA-element—*cis*-acting elements involved in auxin response.

To analyze the potential function of *MtCML* genes, a 1000-bp sequence upstream start codon in the promoter region of *MtCMLs* was analyzed using the PlantCARE. The major *cis*-acting elements related to stress and phytohormone responses are shown in Table S5. ABA (ABRE), MeJA (CGTGA-motif) and auxin (AuxRR-core) response elements were enriched in the promoter of *MtCML*s, while salicylic acid (TCA-motif), gibberellin (GARE-motif), circadian, drought (MBS) and cold (LTR) response elements were also found in the promoters (Figure 5). Seven *MtCMLs* (*MtCML8*, *MtCML20*, *MtCML28*, *MtCML33*, *MtCML35*, *MtCML37* and *MtCML40*) have drought response element, and six *MtCML*s (*MtCML2*, *MtCML6*, *MtCML16*, *MtCML19*, *MtCML33* and *MtCML47*) have cold response element in the promoter (Figure 5). The results indicated that *MtCMLs* may respond to plant hormones and abiotic stresses. *MtActin* was used as an internal control to ascertain if the different *cis*-elements found in the promoters of the *MtCML* genes were or not substantially enriched [41]. Three *cis*-acting elements including two gibberellin responsive elements (GARE-motif and *P-*box), three salicylic acid responsiveness elements (TCA-element) and one light responsiveness (Box 4) were observed in the promoter region of *MtActin* (Table S5), while LTR, ABRE, CGTGA-motif, MBS and AuxRR-core elements were enriched in the *MtCML* promoters regions (Table S5).

### 2.7. Expression Profiles of MtCMLs in Response to Salt, Drought and Cold

**Figure 6:**
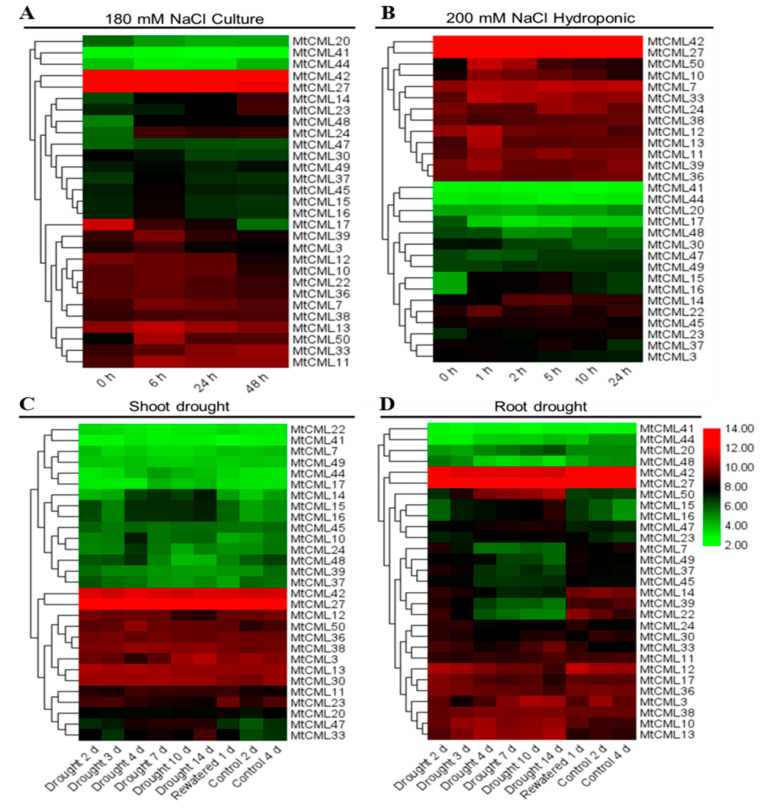
Transcript profile of *MtCML* genes in response to salt and drought stresses. Genome-wide microarray data of *M. truncatula* in different tissues at various developmental stages and the response to drought and salt were retrieved from *M. truncatula* Gene Expression Atlas (MtGEA, https://mtgea.noble.org/v3/). (**A**) Two-day-old seedlings were placed on half strength of MS medium containing 180 mM NaCl for 0, 6, 24 and 48 h, (**B**) Two-week-old seedlings were treated in a nutrient solution containing 200 mM NaCl for 1, 2, 5, 10 and 24 h as hydroponic treatment, while those growing in the nutrient solution as control. **C** and **D**, 24-day-old seedlings growing in soil were stopped watering for drought treatment for 14 d before rewatering, followed by sampling shoot and roots, respectively for analysis of gene expression. The expression levels of the *MtCML* genes are shown as the log_2_-based fluorescence intensity values from the microarray data (MtGEA, https://mtgea.noble.org/v3/).

Gene expression profiles of *MtCMLs* in different tissues under salt, drought and cold stresses were obtained from *M. truncatula* Gene Expression Atlas (MtGEA, https://mtgea.noble.org/v3/). There are 29 *MtCML* genes that have corresponding probe sets in the dataset [42]. *MtCMLs* expression patterns were changed in response to salt and drought stress (Table S3). *MtCML24* and *MtCML50* were significantly upregulated after six hours of salt stress, whereas *MtCML17* was downregulated (Figure 6A). In the hydroponic treatment experiment, *MtCML10*, *MtCML15*, *MtCML16* and *MtCML50* transcripts were upregulated after one hour of salt stress and downregulated at 10 h (Figure 6B). Most of *MtCML* transcripts were unaltered in shoot during drought treatment except for *MtCML33* and *MtCML47*, whose expression was increased significantly (Figure 6C). On the other hand, *MtCML15*, *MtCML16*, *MtCML23*, *MtCML47* and *MtCML50* transcripts in roots were induced by drought treatment, followed by decrease after rewatering, while *MtCML7*, *MtCML14*, *MtCML22*, *MtCML37*, *MtCML39*, *MtCML45* and *MtCML49* transcripts were significantly reduced after drought treatment followed by increase after rewatering (Figure 6D). The results indicated that *MtCML* may participate in drought and salt stress responses.

**Figure 7:**
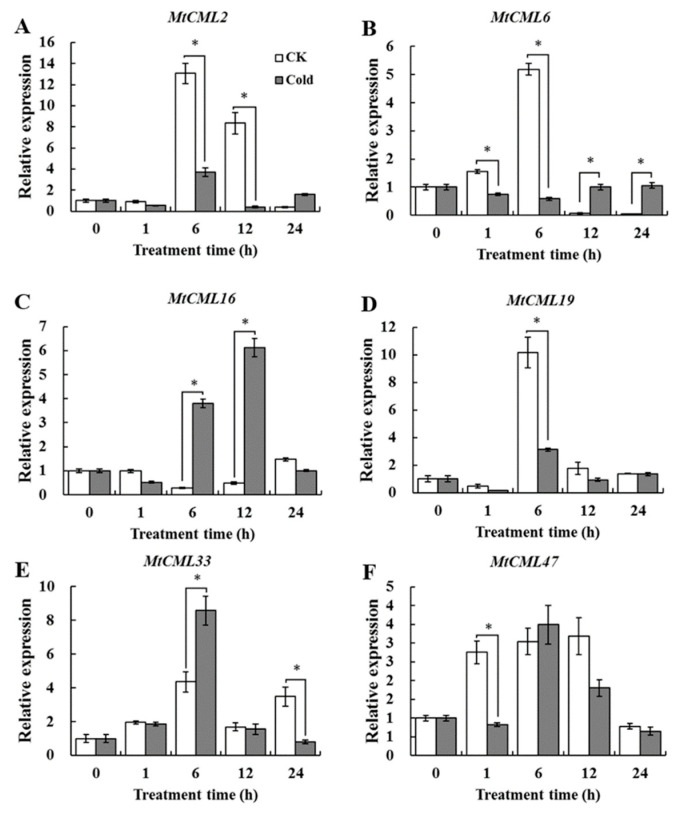
Relative expression of (**A**) *MtCML2*, (**B**) *MtCML6,* (**C**) *MtCML16,* (**D**) *MtCML19,* (**E**) *MtCML33* and (**F**) *MtCML47* in response to cold treatment. Mean values and standard errors of three independent experiments presented. * indicate significant difference between cold treatment and control at *p <* 0.05.

Expression of six *MtCML* genes that have LTR cis-acting element in the promoter regions in response to cold was analyzed (Table S4). *MtCML16* and *MtCML33* transcript levels were induced after six hours of cold treatment, while *MtCML2*, *MtCML6* and *MtCML19* transcripts were significantly reduced (Figure 7). *MtCML47* showed significant downregulation after one hour of cold treatment. The results indicated that *MtCML16*, *MtCML33*, *MtCML2*, *MtCML6*, *MtCML19* and *MtCML47* may participate in cold adaptation in *M. truncatula*.

## 3. Discussion

Camodulin-like proteins play a key role in signal transduction during plant growth and development [3]. A total of 50 *CML* genes were identified in *M. truncatula* in this study, which was same to that in *Arabidopsis* (50 *CML* members) [9], but different from that in rice (32 *CML* genes) [10], grapevine (68 *CML* genes), Chinese cabbage (*79 CML* genes) [12] and tomato (52 *CML* genes) [13]. MtCMLs show extensive variations in gene length, predicted protein size and pI. The varying protein size and pI and hydrophilic property of MtCMLs are consistent with that in CMLs of Chinese cabbage [12,43]. The function of CML members are dependent upon their amino acids sequences, number of EF-hand motifs that are implicated in the Ca^2+^-binding properties and their responses to plant hormones and environmental stresses [8,44]. The number of EF-hand motifs varies among plant species. *Arabidopsis* CML proteins typically possess two to six EF-hand motifs [9], but one to four conserved EF-hand motifs were found in MtCMLs. The diversity of MtCMLs in structure may result in functional diversity. The intron–exon structural analysis showed that most of *MtCML* genes have no intron. This case was consistent with *AtCMLs*, *OsCMLs* and *BrCMLs* [9,10,12].

MtCMLs were classified into eight groups, which is likely similar to those in other plant species [9,12,13]. Phylogenetic analysis of CML proteins revealed that many MtCMLs were homologous to those in *Arabidopsis* and rice. In consistence, most of BrCMLs from *Brassica rapa* were also closely related to their corresponding homologs in *Arabidopsis* and rice [12]. These results suggest that CMLs are conserved among plant species. Gene duplication events are important in the rapid expansion and evolution of gene families [45]. In our study, chromosomal distribution analysis revealed that all *MtCML* genes were randomly scattered on different chromosomes. Homologous gene pairs were found in almost all *MtCMLs*. There are many homologous *CML* genes between the chromosomes in *M. truncatula* and *Arabidopsis.* Whole-genome duplication (WDG) in legumes played a major role in shaping the genome and contributed the raw material for the evolution of nodulation [39,46]; *M*. *truncatula* has undergone high rates of local gene duplication and share an ancient round of gene duplications from other legume species [39,47]. Therefore, WGD and tandem duplication probably participated in driving *MtCML* genes evolution.

The tissue-specific expression patterns of *MtCML* genes are presumed to be associated with their potential biologic roles. Four *MtCMLs* (*MtCML6*, *MtCML13*, *MtCML25* and *MtCML28*) were highly expressed in floral organs, indicating that they are associated with flowering regulation. This can be supported by that AtCML25, the homolog of MtCML25, is involved in pollen germination and pollen tube elongation via regulating Ca^2+^ and K^+^ transmembrane trafficking in pollen grains and pollen tubes in *Arabidopsis* [25]. *MtCML17* and *MtCML22* were highly expressed in roots, indicating that they are associated with roots in *M. truncatula*. However, qRT-PCR showed that *MtCML14* and *MtCML47* were mostly expressed in leaves and seeds rather than roots (Figure 4B–G). The expression difference between qRT-PCR and microarray data may result by the difference of plant growth stages.

The temporal, spatial, and cell type-specific expression of genes are associated with the regulatory elements in the promoter region [48]. Some promoter elements (LTR, ABRE, CGTGA-motif, MBS and AuxRR-core) were enriched multiple times in the promoter regions of *MtCML* genes, but were not found in *MtActin*. It suggested that *MtCML* genes can be involved in regulating multiple hormone responses and abiotic stresses. Previous studies have shown that some *CML* genes participated in the hormonal or abiotic stresses responses when specific *cis*-elements were found in their promoter regions, like *AtCML9* [49], *AtCML20* [36], *AtCML37* [34] and *MtCML40* [40]. This is consistent with our hypothesis. The expression of *CML* genes varies specifically in response to Ca^2+^ signaling and a variety of stress responses, especially abiotic stresses. In the present study, four *MtCML* genes (*MtCML14*, *MtCML17*, *MtCML30* and *MtCML50*) and nearly half of the *MtCML* genes showed extensive responses to salt and drought stresses, respectively. Similar to these results, studies have shown that *AtCML9* was induced under dehydration treatment [2]. Transcripts of *AtCML9*, *AtCML37*, *AtCML38*, *AtCML39* and *OsMSR2* (a rice CML) were up-regulated substantially following salt stress [2,35,50]. Although *MtCML40* is not found in the database, its expression was induced by salt treatment and negatively regulates salt tolerance in *M. truncatula* [40]. Six *MtCML* genes showed response to cold stress. *AtCML24* transcript is induced after cold treatment in *Arabidopsis* [23]. The tomato (*Solanum habrochaites*) *CML* gene, *ShCML44*, was found to improve the tomato tolerance in cold, drought and salinity stresses [51]. These previous studies have shown that CML can play a role in response to salt, drought and low temperature stresses, which is consistent with our qRT-PCR results. These results will help further explore the function of *MtCML* genes in *M. truncatula*.

## 4. Materials and Methods

### 4.1. Identification of CML Genes in M. truncatula

The *M. truncatula* Mt4.0v1 protein sequences were downloaded from Phytozome 12 (https://phytozome.jgi.doe.gov/). The reported CML gene sequences in *A. thaliana* were downloaded from the TAIR database (https://www.arabidopsis.org/) and used as a query to perform BLASTP searching. The NCBI database was used to search the potential CML genes in *M. truncatula*. In addition, the protein sequences of OsCMLs was downloaded from TIGR (http://rice.plantbiology.msu.edu/) and used as queries to search against the *M. truncatula* proteome. To differentiate CaM and CML genes which were homologs in *M. truncatula*, we follow the principle of the major character of EF-hand-containing proteins [13,43] and screen the candidate CML genes. All resulting non-redundant protein sequences were checked for the presence of the entire EF-hand domain by SMART (http://smart.embl-heidelberg.de/) and InterProScan (http://www.ebi.ac.uk/interpro/).

### 4.2. Analysis of Conserved Domain, Gene Structure and Characterization of MtCML Genes

The MtCML protein sequences were analyzed for physical and chemical characteristics, including the molecular weight (MW), theoretical point (pI) and grand average of hydropathicity (GRAVY), using the ProtParam tool of ExPASy (http://web.expasy.org/protparam/). The exon–intron structure analysis of *MtCML* genes was conducted using the TBtools (Toolbox for Biologists) program with default parameters. The conserved motifs were analyzed using the MEME tool (http://meme-suite.org/tools/meme), with the minimum width of motifs as 10, the maximum width of motifs as 40 and the other parameters as default values.

### 4.3. Phylogenetic Relationships of CML Proteins in M. truncatula, Arabidopsis and Rice

The multiple alignments of MtCML proteins were performed using ClustalX with default parameters. Phylogenetic analysis of CMLs in *M. truncatula*, *Arabidopsis* and rice was performed using MEGA X with the maximum-likelihood (ML) method 1000 bootstrap replicates [52].

### 4.4. Chromosomal Locations of MtCML Genes

The sequences of *MtCML* were used to retrieve their chromosomal locations in *M. truncatula* genome databases Phytozome 12 (https://phytozome.jgi.doe.gov/). TBtools software was used for analyze the chromosomal locations and homologous relationship of *MtCML* genes [53]. The Multiple Collinearity Scan toolkit (MCScanX) was used to analyze the gene duplication events [53].

### 4.5. Analysis of cis-Acting Elements of MtCML Genes

A 1000 bp of genomic DNA sequence upstream of the transcriptional start site in each *MtCML* was obtained from the Phytozome database (https://phytozome.jgi.doe.gov), and the PlantCARE database (http://bioinformatics.psb.ugent.be/webtools/plantcare/html/) [54] was used to identify the *cis*-acting elements in the promoter region of *MtCMLs*.

### 4.6. Analysis of Microarray Expression Profile

The genome-wide microarray data of *M. truncatula* in different tissues at various developmental stages and the response to drought and salt were retrieved from *M. truncatula* Gene Expression Atlas (MtGEA, https://mtgea.noble.org/v3/). The transcript data of *MtCMLs* were analyzed using TBtools software. The normalized expression data were used to generate heatmap using the TBtools software [53].

### 4.7. Analysis of Relative Expression of MtCMLs in Different Tissues and Response to Cold Using qRT-PCR

*M. truncatula* (Jemalong) A17 plants were were grown in 15 cm-diameter plastic pots containing a mixture of peat and perlite (3:1, *v/v*) in a greenhouse with temperature ranging from 20 to 28 °C under natural light. Four-week-old plants were exposed to low temperature in a growth chamber at 4 °C for 24 h under 12 h light as cold treatment. Total RNA was extracted using RNAprep pure Plant Kit (TIANGEN, Beijing, China) according to the manufacturer’s instructions. The cDNA was synthesized from 1 μg of total RNA, using the PrimeScript RT reagent Kit with gDNA Eraser (Takara, Japan). *MtCML* genes transcript were analyzed using qRT-PCR in Thermal Cycler Dice Real Time System II (Takara, Japan) following the manufacturer’s instructions. The PCR reaction mix was consisted of diluted cDNA as template, 200 nM forward and reverse primers and 5 μL SYBR Premix Ex Taq (Takara, Dalian, China). Parallel reactions to amplify *actin* were used to normalize the amount of template. Relative expression was calculated by 2^−ΔΔCt^. The expression levels in the control plants without treatment (0 h) were normalized to 1. The *MtActin* gene was used as the internal control. The qRT-PCR primers listed in Table S6 were acquired by the PrimerQuest tool (http://www.idtdna.com/Primerquest/Home/Index).

## 5. Conclusions

A total of 50 *MtCML* genes were identified in *M. truncatula*, which were divided into eight groups. Analysis of intron–exon structure revealed that *CML* gene family is evolutionarily conserved. The functional divergence between subgroups was mainly attributed to changes in the EF-hand domains, which may confers functional differentiation of MtCMLs. Synteny analysis showed that WGD and tandem duplication probably participated in driving *MtCML* genes evolution. Expression profile analysis showed that most of *MtCML* genes are expressed in leaves, flowers and roots and had a tissue and organ expression preference. Many *cis*-acting element responsive to plant hormones, such as ABA, MeJA and auxin were enriched in the promoter of *MtCML*s, while salicylic acid (TCA-motif), gibberellin (GARE-motif), circadian, drought (MBS), and cold (LTR) response elements were found in the promoter of some *MtCMLs*. Many *MtCMLs* were regulated by drought and salt stresses. qRT-PCR analysis showed that *MtCML16* and *MtCML33* were upregulated, *MtCML2*, *MtCML6*, *MtCML19* and *MtCML47* were downregulated in response to cold treatment. Our results provide new information for further understanding of the structure, evolution and function of *MtCML* gene family.

## Figures and Tables

**Table 1:** Information on MtCML proteins.

Name	Locus ID	CDS (bp)	A.A.	Mw (KDa)	pI	GRAVY
MtCML1	Medtr1g019600.1	303	101	11.57	8.73	−0.412
MtCML2	Medtr1g019610.1	294	98	11.29	7.75	−0.744
MtCML3	Medtr1g019640.1	288	96	10.91	9.01	−0.747
MtCML4	Medtr1g019660.1	288	96	10.88	6.27	−0.589
MtCML5	Medtr1g030440.1	504	168	19.17	4.26	−0.137
MtCML6	Medtr1g032070.1	597	199	21.91	4.36	−0.532
MtCML7	Medtr1g041285.1	453	151	17.03	4.01	−0.401
MtCML8	Medtr1g046950.1	429	143	16.04	4.54	−0.675
MtCML9	Medtr1g047100.1	426	142	15.75	4.43	−0.537
MtCML10	Medtr1g076650.1	453	151	17.16	4.07	−0.498
MtCML11	Medtr2g086560.1	798	266	29.98	6.59	−0.703
MtCML12	Medtr2g098890.1	636	212	24.24	4.77	−0.319
MtCML13	Medtr3g067610.1	417	139	15.71	4.99	−0.862
MtCML14	Medtr3g088655.1	495	160	18.47	4.35	−0.481
MtCML15	Medtr3g089070.1	423	141	16.03	4.55	−0.474
MtCML16	Medtr3g089090.1	423	140	15.90	4.55	−0.491
MtCML17	Medtr3g109320.1	540	180	20.65	4.58	−0.475
MtCML18	Medtr4g067270.1	453	151	17.08	4.37	−0.546
MtCML19	Medtr4g082050.1	543	144	16.32	4.38	−0.175
MtCML20	Medtr4g086260.1	591	197	22.73	8.42	−0.704
MtCML21	Medtr4g103630.1	423	141	16.00	4.43	−0.494
MtCML22	Medtr4g112460.1	510	170	18.74	4.5	−0.087
MtCML23	Medtr4g115170.1	681	226	25.26	4.46	−0.219
MtCML24	Medtr4g127560.1	600	200	23.04	4.48	−0.368
MtCML25	Medtr5g008695.1	348	115	13.15	4.38	−0.63
MtCML26	Medtr5g008705.1	393	131	14.90	4.68	−0.706
MtCML27	Medtr5g011850.1	444	148	16.59	4.64	−0.418
MtCML28	Medtr5g011920.1	195	65	7.37	6.71	−0.17
MtCML29	Medtr5g017510.1	570	190	21.24	6.63	−0.584
MtCML30	Medtr5g017550.1	567	189	21.16	6.64	−0.661
MtCML31	Medtr5g017560.1	570	190	21.24	6.63	−0.584
MtCML32	Medtr5g025690.1	603	201	23.26	4.34	−0.325
MtCML33	Medtr5g079340.1	420	140	15.78	4.56	−0.689
MtCML34	Medtr6g007613.1	513	171	19.80	4.86	−0.88
MtCML35	Medtr6g023460.1	429	143	15.95	4.37	−0.32
MtCML36	Medtr6g079570.1	525	175	19.31	4.35	−0.584
MtCML37	Medtr7g011010.1	609	203	23.61	4.46	−0.307
MtCML38	Medtr7g074020.1	516	172	19.77	4.84	−0.961
MtCML39	Medtr7g074240.1	537	179	20.08	4.45	−0.357
MtCML40	Medtr7g075040.1	588	196	22.01	4.71	−0.352
MtCML41	Medtr7g089760.1	459	153	16.97	4.16	−0.472
MtCML42	Medtr7g090450.1	651	217	23.86	4.67	−0.478
MtCML43	Medtr7g451050.1	717	239	27.95	5.08	−0.455
MtCML44	Medtr8g036075.1	597	199	22.55	5.32	−0.36
MtCML45	Medtr8g066630.1	495	165	18.40	4.6	−0.373
MtCML46	Medtr8g069915.1	510	170	18.77	4.26	−0.242
MtCML47	Medtr8g070510.1	684	228	26.11	4.65	−0.442
MtCML48	Medtr8g078270.1	567	189	21.51	5.01	−0.66
MtCML49	Medtr8g105230.1	573	191	20.77	4.35	−0.523
MtCML50	Medtr8g107110.1	498	166	18.22	4.31	−0.553

Note: A.A.—number of amino acids sequence; pI—theoretical isoelectric point of proteins; MW—theoretical molecular weight of proteins; GRAVY—grand average of hydropathicity.

## Supplementary Materials

The following are available online at https://www.mdpi.com/1422-0067/21/19/7142/s1. Table S1. The CML protein sequence in *Medicago truncatula*. Table S2. The expression pattern of *MtCML* genes in different developmental tissues of *Medicago truncatula*. Table S3. The expression level of the *MtCML* genes under salt and drought stresses from the microarray data. Table S4. The relative expression of *MtCML* genes in cold treatment. Table S5. The promoter information of *MtCML* genes. Table S6. Sequences of primers used in qRT-PCR. Table S7. The percentages of identity between pairs of paralogous MtCML proteins.
